# Antiproliferative effect of mifepristone (RU486) on human neuroblastoma cells (SK-N-SH): *in vitro* and *in vivo* studies

**DOI:** 10.1590/1414-431X202010067

**Published:** 2020-10-07

**Authors:** L.A. Casulari, D. Dondi, G. Pratesi, F. Piva, M. Milani, M. Piccolella, R. Maggi

**Affiliations:** 1 Serviço de Endocrinologia, Hospital Universitário de Brasília, Universidade de Brasília, Brasília, DF, Brasil; 2 Department of Pharmaceutical Sciences, Universitè degli Studi di Milano, Department of Pharmaceutical SciencesUniversitè degli Studi di MilanoItalyItaly; 3 Department of Experimental Oncology, IRCCS, Istituto Nazionale Tumori, Milano, Italy; 4 Department of Pharmacological and Biomolecular Sciences, Universitè degli Studi di Milano, Department of Pharmacological and Biomolecular SciencesUniversitè degli Studi di MilanoItalyItaly; 5 ASST Ospedale di Lecco, Lecco, Italy

**Keywords:** Mifepristone, RU486, Cell proliferation, Neuroblastoma, Nude mice, Tumor cell growth

## Abstract

RU486 (mifepristone), a glucocorticoid and progesterone receptor antagonist, has been reported to exert antiproliferative effects on tumor cells. Experiments were performed to analyze the effects of RU486 on the proliferation of the human neuroblastoma, both *in vitro* and *in vivo*, using the human neuroblastoma SK-N-SH cell line. The exposure *in vitro* of SK-N-SH cells to RU486 revealed a dose-dependent inhibition of ^3^H-thymidine incorporation due to a rapid but persistent inhibition of MAPKinase activity and ERK phosphorylation. A significant decrease of SK-N-SH cell number was evident after 3, 6, and 9 days of treatment (up to 40% inhibition), without evident cell death. The inhibitory effect exerted by RU486 was not reversed by the treatment of the cells with dexamethasone or progesterone. Moreover, RU486 induced a shift in SK-N-SH cell phenotypes, with an almost complete disappearance of the neuronal-like and a prevalence of the epithelial-like cell subtypes. Finally, the treatment with RU486 of nude mice carrying a SK-N-SH cell xenograft induced a strong inhibition (up to 80%) of tumor growth. These results indicated a clear effect of RU486 on the growth of SK-N-SH neuroblastoma cells that does not seem to be mediated through the classical steroid receptors. RU486 acted mainly on the more aggressive component of the SK-N-SH cell line and its effect *in vivo* was achieved at a concentration already used to inhibit oocyte implantation.

## Introduction

Neuroblastoma is the most common solid extracranial tumor that occurs in a child's first year of life. It is more common in boys than in girls, but the basis for this prevalence is unclear. There is minimal variation in incidence in relation to race and geographic location. However, in North America, it is more common to have the most malignant form in individuals with African ancestry than in those with European ancestry (1,2).

Clinical and biological heterogeneity occurs in the presentation of these tumors. Thus, they can be classified into three risk categories: low, intermediate, and high. The factors that influence the prognosis include tumor stage, age at diagnosis, histopathology of the tumor, DNA index (ploidy), and the presence or absence of MYCN amplification. Tumors considered to be of high risk often have amplification of the MYCN oncogene and segmental chromosomal changes. The prognosis of children with low and intermediate risks are excellent, with a survival rate of up to 90%. Many of them even have spontaneous regression. However, children with high-risk tumors have a poor prognosis and a long-term survival rate of less than 50%. Despite the several treatments available, there are still no good results in children with the most severe type of neuroblastoma (1,2).

The presence of hormone receptors in neoplastic cells, which can interfere with cell growth, opens new perspectives in the control of tumors (2). Thus, hormones such as progesterone and cortisol have been reported to influence cell growth in various types of tumors (3,4). For instance, it has been reported that high doses of natural progesterone inhibit the growth of human neuroblastoma cells (SK-N-AS) both *in vitro* and *in vivo* by suppression of cell proliferation and induction of apoptosis (4). Moreover, we have previously demonstrated that dexamethasone may block the migration of neuroblastoma cell line SK-N-SH (5).

RU486 [17β-hydroxy-11β-(4-dimethylamino-phenyl-1)-17-(prop-1-ynyl) estra-4,9-dien-3-one, mifepristone] is a potent blocker of progesterone and cortisol receptors and has been used in the long-term treatment of meningiomas (6). Antiproliferative effects of RU486 have been described for SKOV-3 and IGROV-1 ovarian cancer cells *in vitro* and for IGROV-1 cells *in vivo*, implanted in nude mice (7). Inhibition of cell growth induced by RU486 metabolite metapristone was also observed in melanoma B16F10 cells *in vitro* and C57BL/6 cells xenograft *in vivo* (8). RU486 also improves the action of temozolomide in blocking the growth of C6 glioma cells *in vitro* and *in vivo* (9). Finally, discrepancies in the antiproliferative effects of RU486 in *in vitro* and *in vivo* models have also been described. In a metastatic prostate cancer model, the effect of RU486 to improve the antiproliferative action of docetaxel or enzalutamide *in vitro* was not observed when these cells were implanted in athymic nude mice (10). Moreover, the development of resistance to RU486 in melanoma cells implanted in nude mice has also been described (11).

On the other hand, we have previously shown that antiproliferative effects of RU486 on the human neuroblastoma SK-N-SH cells *in vitro* is not blocked by either dexamethasone or progesterone (12), suggesting that mifepristone would exert its antiproliferative action through a mechanism that does not involve classical steroid hormone receptors. Progesterone receptor-independent antiproliferative effects of both RU486 and progesterone have been observed in other experimental models *in vivo* and *in vitro* (13-16).

In the present study, the possible effects of RU486 on the proliferation of human neuroblastoma SK-N-SH cell line have been investigated by *in vitro* and *in vivo* experiments.

## Material and Methods

### Chemicals

RU486 [17β-hydroxy-11β-(4-dimethylamino-phenyl-1)-17-(prop-1-ynyl) estra-4,9-dien-3-one] (mifepristone) was kindly supplied by Roussel-Uclaf (France). Progesterone was obtained from Sigma-Aldrich Co. (USA) and dexamethasone (Dex) was obtained as a commercial preparation (Soldesam, LFM, Italy). Stock solutions of RU486 and progesterone were made up in dimethyl sulfoxide (DMSO) and stored at +4°C. The final concentration of DMSO in the culture medium was 0.1%; in preliminary experiments, this concentration proved not to influence cell proliferation/survival. [methyl-^3^H]thymidine (25 Ci/mmol) was obtained from GE Healthcare (USA). MTT (3-[4,5-dimethyl(thiazol-2-yl)-3,5-diphenyl] tetrazolium bromide) was from Fluka (Switzerland). Fetal calf serum (FCS) was obtained from Gibco (Scotland). Minimal essential medium (MEM), non-essential amino-acids, and sodium pyruvate was purchased from Biochrom KG (Germany).

### Cell cultures

Human neuroblastoma cell line SK-N-SH was supplied by American Type Culture Collection (USA). SK-N-SH cells were routinely grown in MEM-EARLE supplemented with 10% fetal bovine serum (FBS), non-essential amino-acids, and antibiotics (100 IU penicillin, 100 µg/mL streptomycin) (Biochrom KG).

Cells were grown at 37°C in a 5% CO_2_ humid atmosphere. Culture medium was replaced every 2 days. For experiments using synthetic steroids and hormones, charcoal-stripped FBS was used to reduce its steroid contents.

### [^3^H]-Thymidine incorporation

Cells were plated in 24-well plates at a density of 50,000 cells/well in 1 mL of medium. The cells were then cultured for 2 days without changing the culture medium. On day 3, the medium was replaced, and cells were treated with increasing concentrations of RU486. After a further 20 h, [^3^H]-thymidine was added to the culture medium (1 µCi/mL), and the plates were incubated for an additional 4 h. Cells were subsequently washed with pre-warmed PBS, and then, 1 mL of cold 10% trichloroacetic acid was added. The cells were dissolved by the addition of 200 µL of 6 M NaOH and counted in 7 mL Instagel scintillation cocktail (Packard Instruments, Italy). Three wells were used for each sample.

### Cell survival evaluated by colorimetric assay (MTT)

MTT assay is based of the conversion of the yellow tetrazolium salt (MTT) to a blue-purple formazan dye, an event that occurs only in the presence of living cells through the action of a mitochondrial succinic dehydrogenase (17). Neuroblastoma cells were seeded in 24-well plates at a density of 10,000 cells/well. On the following day (day 0) and subsequently every two days, the media, containing DMSO 0.1% or RU486, were changed. MTT (1 mg/mL) was dissolved in culture medium without FBS and phenol red and filtered through a 0.22 µm filter. The media were substituted with 1 mL/well of the pre-warmed MTT solution. The cells were then incubated for 2 h at 37°C, and the MTT solution was replaced by 1 mL isopropyl alcohol to solubilize the formazan crystals. The formation of formazan by the cells was measured spectrophotometrically at 560 nm. All assays were done in triplicate.

### MAPK activity

SK-N-SH cells were seeded (1.5-2.0×10^6^) in culture medium with 0.1% FBS and preincubated for 10 min with RU486 before the addition of 20% FBS, used to stimulate MAPK activity. After 5 min incubation, the culture medium was decanted and the cells were collected in 0.5 mL of homogenizer buffer (50 mM 0-glycerophosphate, pH 7.3, 1.5 mM EGTA, 1 mM EDTA, 1 mM dithiothreitol (DT"), 0.1 mM sodium vanadate, 1 mM benzamidine, 10 pg/mL aprotinin, 10 pg/mL leupeptin, and 2 pg/mL pepstatin A), as described previously (18,19). After sonication, the samples were centrifuged (100,000 *g*, 10 min, 4°C) and the resulting supernatant (cytosolic fraction) was fractionated on DEAE cellulose columns. The activity of MAPK was determined by measuring the incorporation of phosphate in myelin basic protein (MBP, Sigma Aldrich Co.) in the presence of 32P ATP (2 Ci/sample; specific activity 3000 Ci/mmol; GE Healthcare), as described previously (18,19). Levels of phosphate incorporation measured in the absence of substrate were subtracted from levels obtained in the presence of substrate to correct for nonspecific phosphorylation. These values were less than 22% of the specific phosphorylation.

### Immunoblot analysis

In a first series of experiments, SK-N-SH cells, plated in 10-mm dishes in serum-free medium, were treated for 2, 5, and 20 min with RU486 (10 μM). In a second series of experiments SK-N-SH cells were treated for 48 h and 6 days with RU486 (10 μM) and Dex (0.1 μM), as indicated. SK-N-SH cells were harvested in RIPA buffer added with protease inhibitors (0.4 mg/mL AEBSF, 2 µg/mL leupeptin, and 2 µg/mL pepstatin), centrifuged (9000 *g*, 10 min, 4°C), and washed in PBS. Protein concentration was determined using the Bradford assay. Equal amount of protein (50 µg) were resolved on 10% SDS-polyacrylamide gel electrophoresis (SDS-PAGE), for 1.5 h at 110 Volts. Proteins were blotted using a transfer apparatus (Trans-Blot semi-dry, Bio-Rad, Italy). The membrane was washed with 10 mM Tris-HCl, 150 mM NaCl, 0.1% Tween 20 (TBST) for 30 min, immersed in a blocking solution with TBST and 5% (w/v) dry skimmed milk, and then incubated with a diluted solution of the primary antibody at 4°C overnight. For ERK1/2 analyses, we used 1:100 mouse monoclonal antibodies against P-ERK1/2 (E-4, Santa Cruz Biotechnology, USA), and 1:1000 rabbit polyclonal antibody against ERK1/2 (K-23, Santa Cruz Biotechnology). After incubation, the membranes were washed and incubated for 1 h with a second antibody conjugated with peroxidase (1:10.000). Immunoreactive bands were visualized using the enhanced chemiluminescence detection kit reagents (ECL Plus Western Blotting Detection System, GE Healthcare).

### Actin staining

Subconfluent SK-N-SH cells were plated in 24-well plates on 10-mm poly-L-lysine coated glasses (50,000 cells/well in 500 µL MEM-EARLE medium with 10% FBS) and treated for 6 days with RU486 (10 µM). The organization of the cytoskeleton of SK-N-SH cells was analyzed through the use of FITC-labeled phalloidin. Briefly, cells were fixed in 4% paraformaldehyde and the cell membranes were permeabilized with 0.1% Triton X-100, incubated with FITC-labeled phalloidin (0.2 μg/mL, Sigma Aldrich Co.) at 37°C for 10 min. Samples were observed under a confocal laser-scanning microscope (MicroRadiance 2100, Bio-Rad) mounted on an inverted microscope (Nikon Eclipse 300). The data obtained were compared to those derived from cells treated with DMSO (0.1%) as controls.

### Animals and *in vivo* experiments

The studies made on the animals were approved by IRCCS Istituto Nazionale Tumori, Italy.

Athymic nude mice (5-6 weeks old; Charles River, Italy) were maintained in an aseptic room, with laminal airflow, temperature of 24-26°C, and humidity of 50% under the guidelines complying with the national laws for the use of laboratory animals. Before any invasive manipulation, mice were anesthetized with a mixture of ketamine (25 mg/mL) and xylazine (5 mg/mL). Primary xenografts were obtained by injecting 40×10^6^ SK-N-SH cells subcutaneously (*sc*).

SK-N-SH-derived tumor was maintained *in vivo* by serial subcutaneous passages of tumor tissue fragments (about 2×2×5 mm) in healthy mice, as previously described (20). Tumor fragments were implanted at day 1 and tumor growth was monitored for each experimental group at the time indicated by measuring the average tumor diameter (two perpendicular axes of the tumor were measured by a Vernier caliper). The volume of the tumor is reported in mm^3^ according to the formula 4/3πr^3^.

Tumor weight (TW) was calculated according to the formula TW (mg) = tumor volume (mm^3^) = d^2^ × D/2, where d and D are the shortest and the longest diameters, respectively. Drug treatment started on day 1, shortly after tumor implant.

The RU486 solution used for the treatment was prepared with 110 µL of benzyl alcohol in a test tube containing 11 mg of RU486 and then 2.2 mL of sesame oil was added slowly while mixing well. The solution contained 1.5 mg/mL and was conserved protected from light and in ambient temperature. A solution containing benzyl alcohol and sesame oil in the same proportion was used for the treatment of the control animals. RU486 was delivered *sc*, in a volume of 20 mL/kg of body weight, at the dose of 15 mg/kg (about 0.3-0.5 mg/mouse). The treatments of the animals were started immediately after tumor implantation and repeated twice a week for a total of six treatments (20 days). After that period, the treatment was suspended and the growth of the tumor was observed up to 29 days after the inoculation of the tumor.

At the time indicated, mice were sacrificed by carbon dioxide inhalation and tumor mass was excised and weighed. All animals were subjected to an accurate necropsy and portions of tumors were fixed in 10% buffered formalin, embedded in paraffin, sectioned in 10 µm-sections and processed with standard hematoxylin-eosin staining for routine histological examination.

### Statistical analysis

The data from all experiments were analyzed by ANOVA and adequate *post hoc* tests. P<0.05 was considered significant.

## Results

### Effects of RU486 on SK-N-SH cells *in vitro*

**Figure 1:**
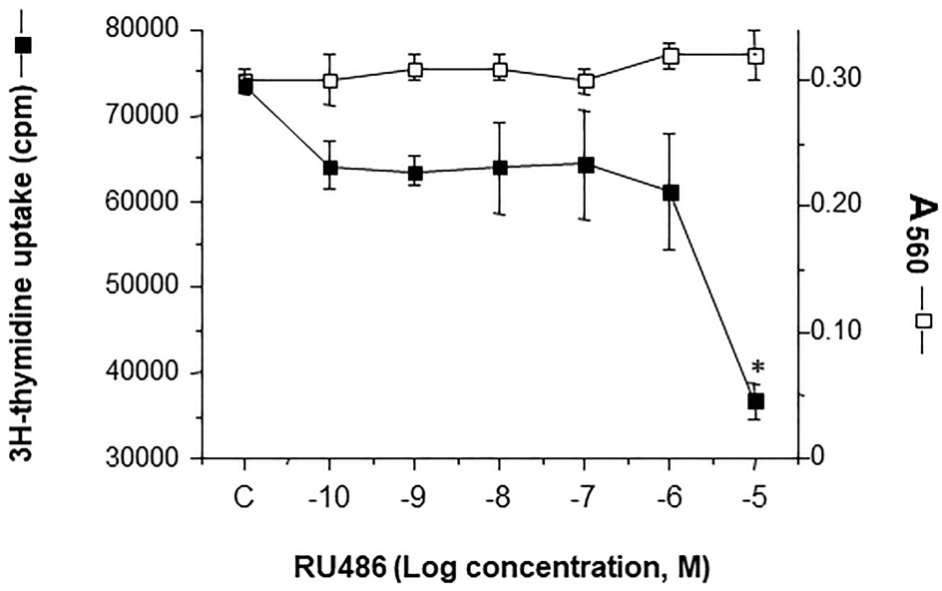
Effect of a 24-h treatment of SK-N-SH cells with RU486 on [3H]-thymidine incorporation and cell vitality (MTT assay). The amount of cells is reported as count per minute (cpm) or absorbance at 560 nm. Data are reported as means±SD; *P<0.05 *vs* control (C, DMSO).

The effect of RU486 on [^3^H]-thymidine incorporation in SK-N-SH cells is reported in Figure 1. The 24-h treatment with RU486 showed a dose-related decrease of [^3^H]-thymidine with a maximum effect at 10 µM concentration of the drug. The decrease of [^3^H]-thymidine incorporation was not due to a decrease of cell number, measured by MTT assay, thus excluding a toxic effect of the treatment.

**Figure 2:**
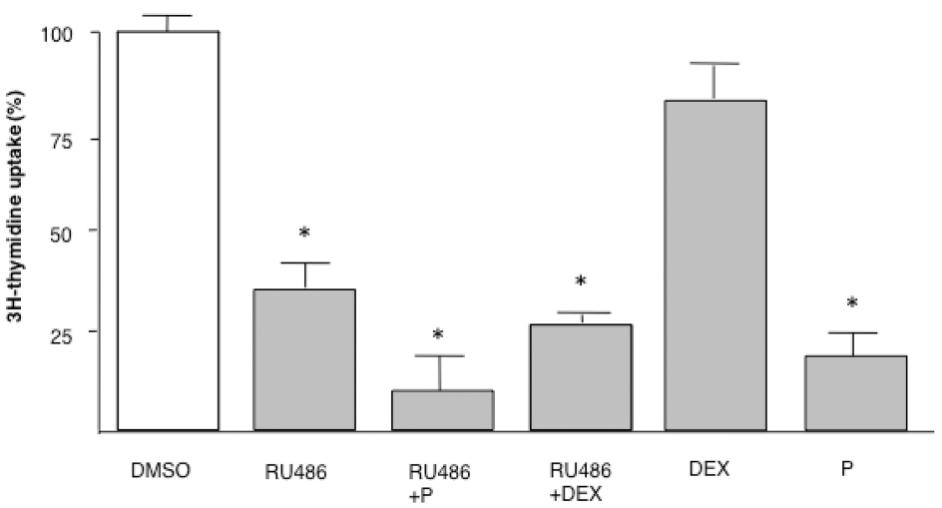
Dexamethasone (DEX) and progesterone (P) did not affect the inhibitory effect of RU486 on [3H]-thymidine incorporation in SK-N-SH human neuroblastoma cells. SK-N-SH cells were treated with RU486 (10 µM), DEX (10 µM), or P (10 µM) alone or in combination for 24 h. Data are reported as the mean±SD of three experiments done in triplicate. *P<0.05 *vs* DMSO (ANOVA).

The inhibition of the [^3^H]-thymidine uptake after 24-h treatment with 10 µM RU486 was not reversed by the presence of an equal concentration of progesterone or the glucocorticoid against dexamethasone (Figure 2). Dexamethasone alone was ineffective: progesterone induced a significant inhibitory effect and slightly potentiated the RU486 effect, through a possible toxic action.

**Figure 3:**
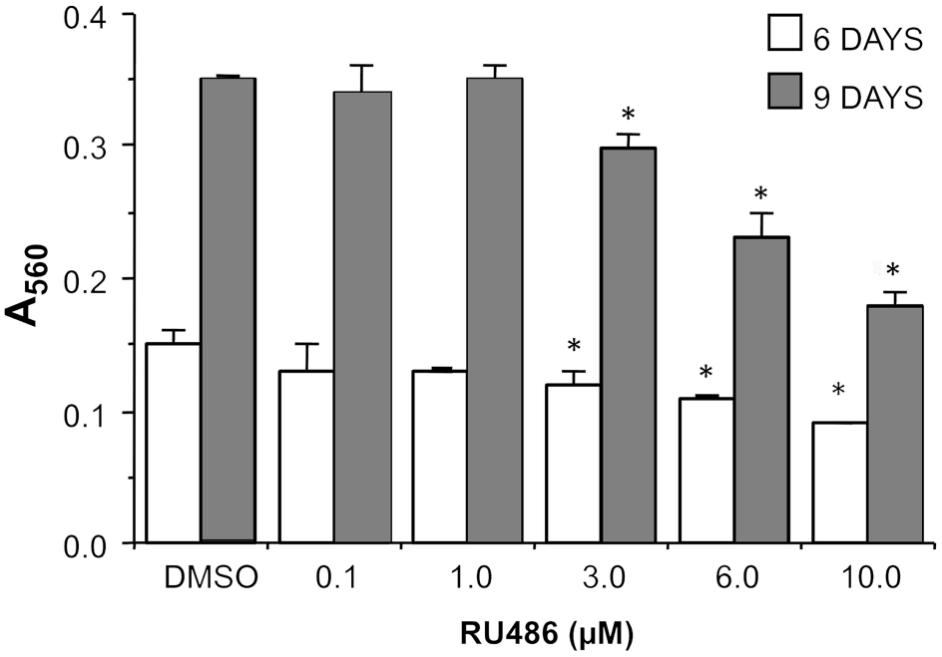
Dose-dependent inhibition of SK-N-SH cell viability after the exposure to RU486 for 6 and 9 days. The results are reported as the mean±SD of absorbance at 560 nm of three experiments done in triplicate. *P<0.05 *vs* DMSO (ANOVA).

A prolonged exposure to RU486 showed a dose-dependent decrease of viable SK-N-SH cells, measured by MTT assay, that appeared to be significant at 6 and 10 µM concentration (Figure 3). The maximal inhibition achieved after 6 days of treatment was 30% and those observed after 9 days was 50% (calculated IC50 5.1±0.2 µM). The effect observed at 10 µM RU486 was confirmed with cell counting (data not shown). Moreover, non-detachment or death of cells were observed along the treatment period, suggesting a cytostatic rather than cytotoxic effect of the compound.

**Figure 4:**
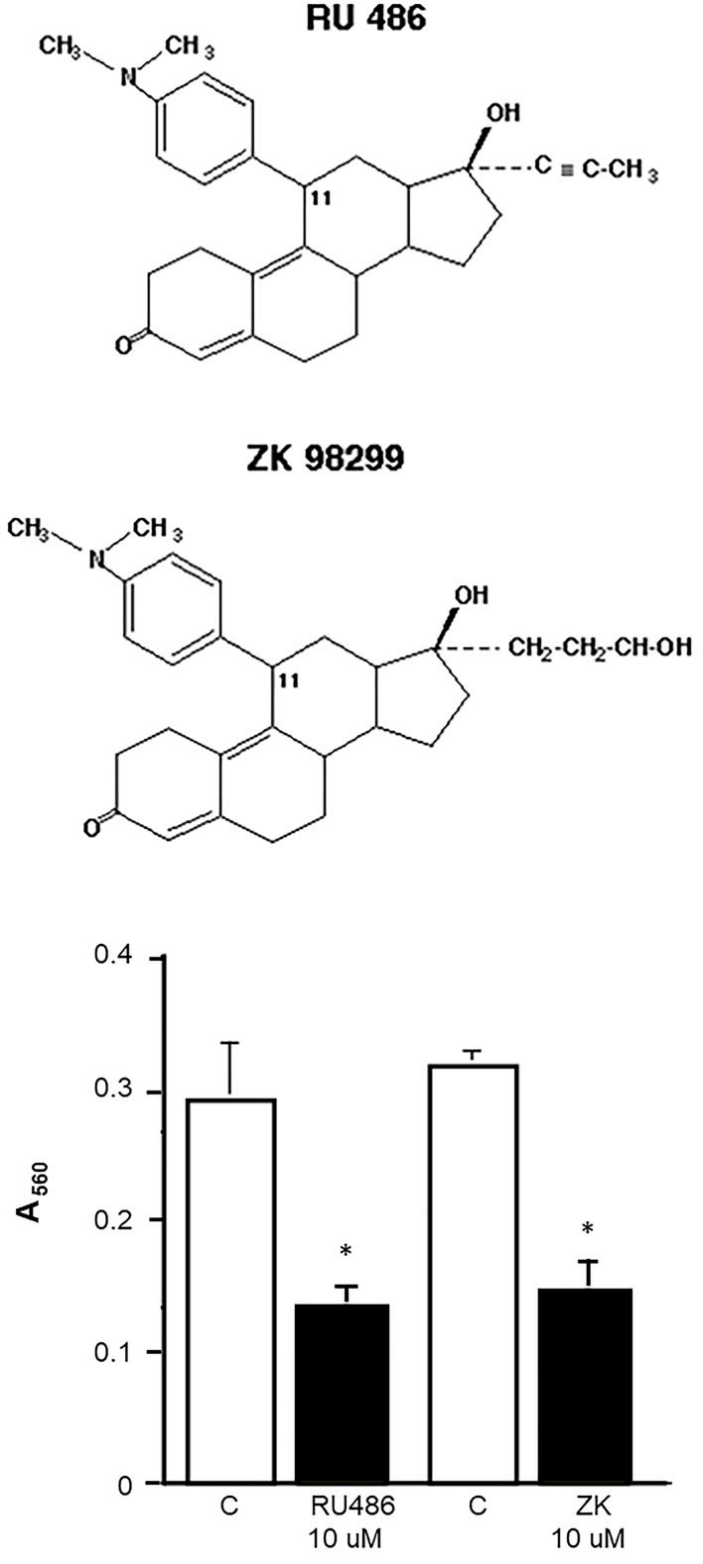
Chemical structure of antiprogestin/glucocorticoid antagonists RU486 and ZK98299. The graph shows the viability of SK-N-SH cells exposed for 9 days to 10 mM concentration of RU486 and ZK98299. Data are reported as means±SD of absorbance at 560 nm. *P<0.05 *vs* control (C, DMSO) (Student’s *t*-test).

In order to also verify the specificity of the effect of RU486, we tested the effect of the exposure of SK-N-SH cells to another synthetic steroid, ZK98299 (onapristone). This molecule is structurally similar to RU486 but it is considered to be a pure progesterone receptor antagonist with reduced or null effect on glucocorticoid receptors (21). The exposure of SK-N-SH cells for 9 days with 10 µM ZK98299 reduced the number of viable cells with the same efficacy of RU486 (Figure 4).

**Figure 5:**
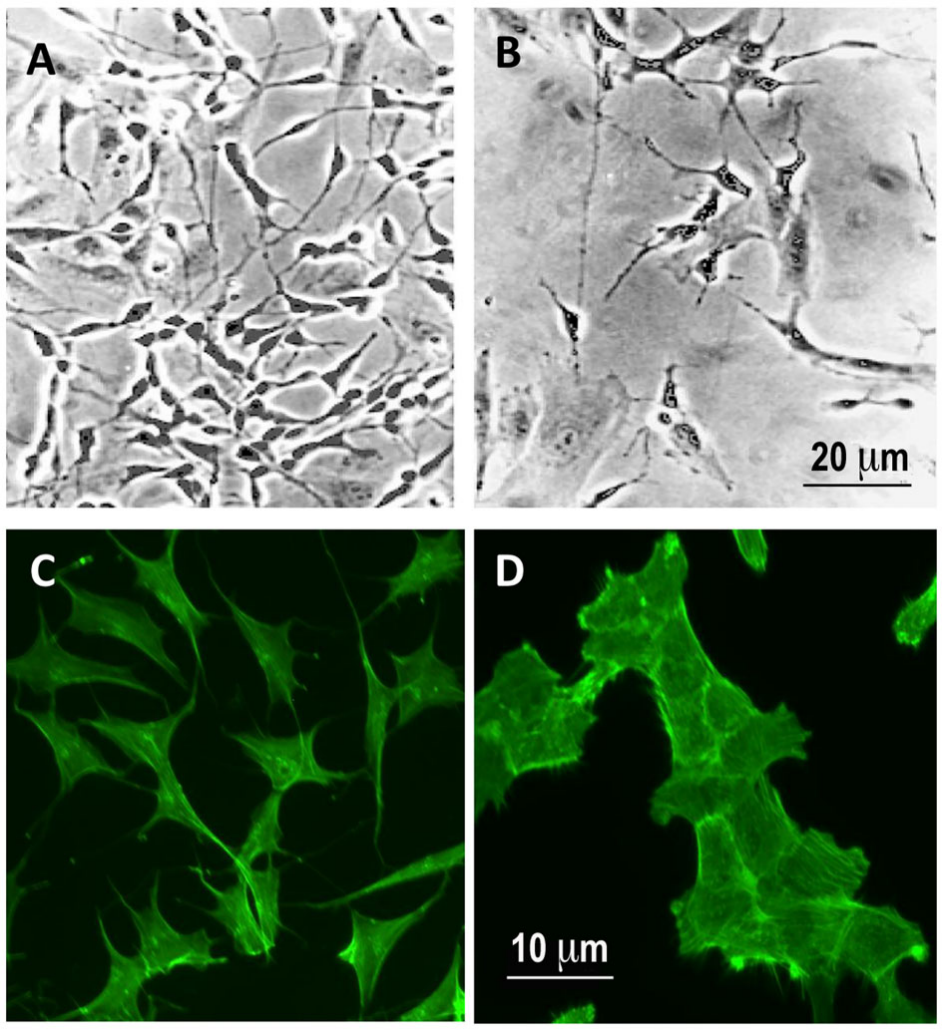
Exposure to 10 mM RU486 for 6 days induced a modification of the cell population toward a fibroblast-like phenotype. Phase contrast (**A** and **B**) and fluorescein-phalloidin staining (**C** and **D**) of SK-N-SH cells treated for 6 days with vehicle (DMSO 0.1%; **A** and **C**) and 10 mM RU486 (**B** and **D**). Magnification bars: 20 μm (**A** and **B**) and 10 μm (**C** and **D**).

Exposure to 10 µM RU486 for 6 days induced a change of the phenotype of SK-N-SH cell line with an apparent prevalence of fibroblast-like phenotype compared to control SK-N-SH cells that showed a main fusiform shape (Figure 5A and B). This observation was confirmed by staining the cells with fluorescein-phalloidin that showed a dispersed fluorescence of the complex phalloidin-F-actin in the cytoplasm of control cells and a fibroblast-like actin distribution in RU486-treated SK-N-SH cells (Figure 5C and D).

### Effects of RU486 on MAPK and ERK activation

**Figure 6:**
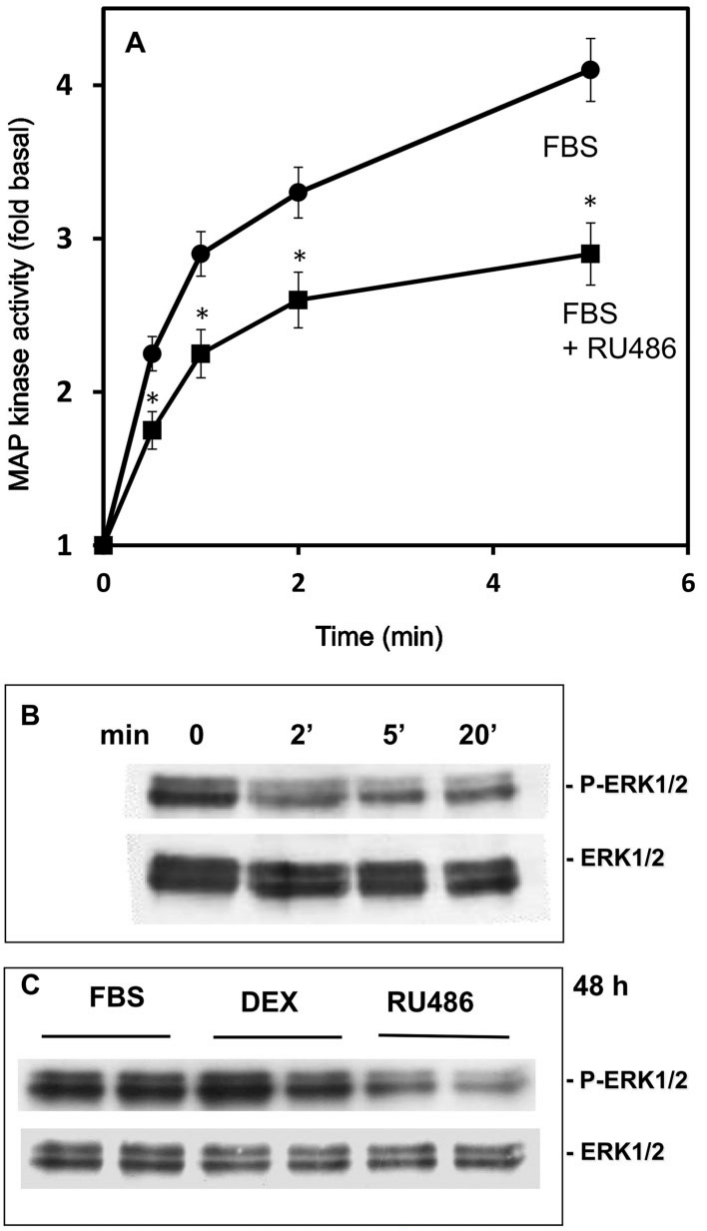
Effect of the exposure of SK-N-SH cells to 10 µM RU486 on MAP kinase activity (**A**), and p-ERK and total ERK immunoreaction by a time course observation (**B**). **C**, p-ERK and total ERK immunoreaction measured after a 48-h exposure to FBS, 10 µM RU486, or 10 µM dexamethasone (DEX); cells exposed to FBS and dexamethasone were analyzed as control treatments. The cell extracts were subjected to western blot analysis to detect phosphorylated and total ERK. Data in panel A are reported as means±SD. *P<0.05 *vs* FBS (ANOVA).

In the attempt to investigate the mechanism of action of RU486, experiments were carried out to study the major intracellular pathways involved in cell proliferation (MAPK and ERK). First, MAPK activity was measured in the first 5 min after the exposure of SK-N-SH cells to FBS in the absence and in the presence of 10 µM RU486; the results indicated that exposure to the drug induced a rapid significant decrease in the MAPK activation induced by FBS (Figure 6A). Then, immunoblotting experiments on extracts of SK-N-SH cells exposed for 2, 5, and 20 min to RU486 (10 µM) were also done. RU486 actually decreased ERK phosphorylation; this effect was clearly evident after 2 min of treatment and was maintained up to 20 min (Figure 6B). Moreover, we studied the effect of treatment for 48 h with RU486 (10 µM) or Dex (0.1 µM), used as treatment control, on FBS-stimulated ERK phosphorylation in SK-N-SH cells (Figure 6C). Immunoblot analysis of SK-N-SH extracts also showed that only RU486 produced a decrease of ERK phosphorylation also after 48 h of treatment, while Dex was completely ineffective at any time considered.

### Effects of RU486 on SK-N-SH cells *in vivo*

**Figure 7:**
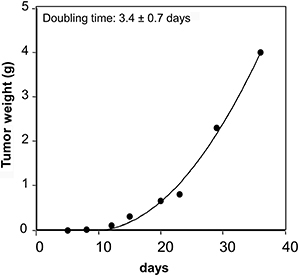
SK-N-SH cells (40×10^6^ cells) were inoculated by *sc* injection in athymic nude mice and left to grow for 35 days. The results are representative of 2 experiments done in triplicate.

The SK-N-SH-derived tumor showed exponential growth with a doubling time of 3.4 days (Figure 7). The tumor was successfully propagated for 5-10 generations by *sc* implantation of the original tumor fragments (see Materials and Methods) and used for further experiments.

**Figure 8:**
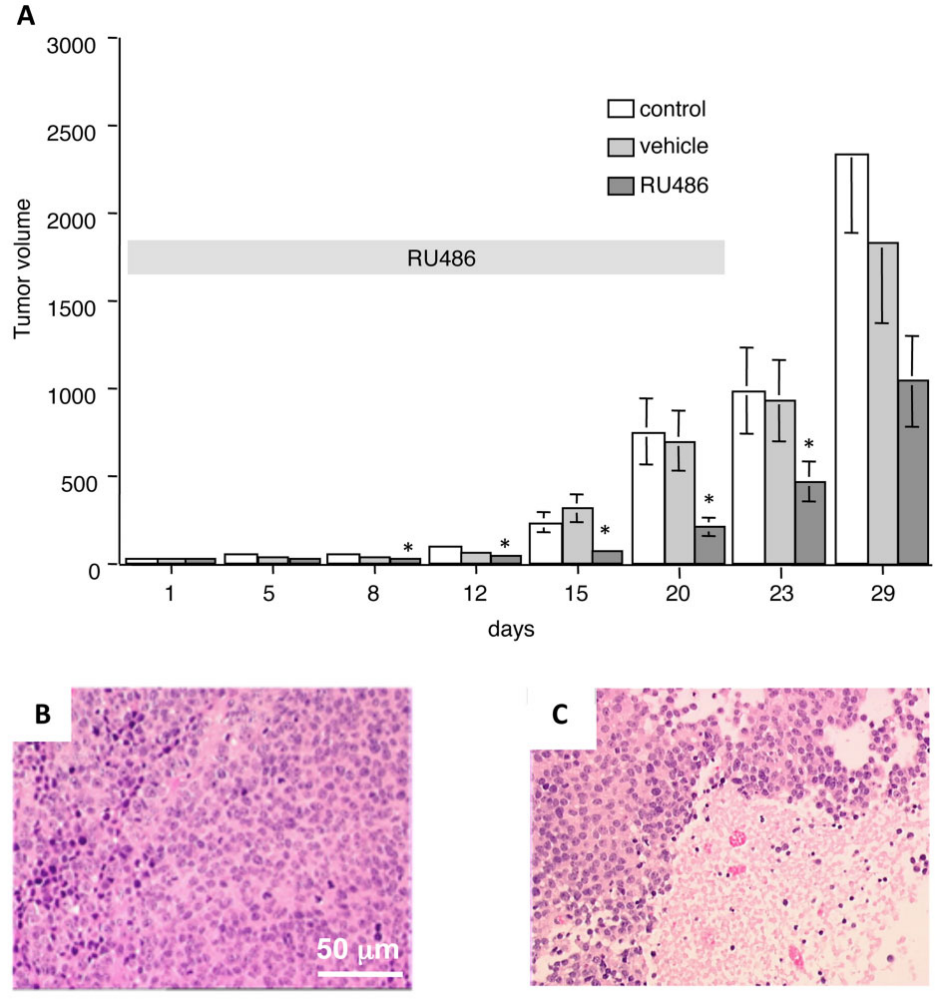
Effect of RU486 on the growth of the SK-N-SH cell-derived neuroblastoma xenograft in nude mice. **A**, Nude mice implanted with fragments of the SK-N-SH cell derived-tumors (1 mm^2^) were treated with RU486 (15 mg/kg, two treatments/week) from day 1 to day 20 (horizontal gray band) and sacrificed on day 29. The results represent the mean of three independent experiments done in triplicate. Hematoxylin-eosin staining of 10-μm sections of control (**B**) or RU486-treated (**C**) xenografted tumors (magnification bar: 50 μm). Data are reported as means±SD. *P<0.05 *vs* vehicle (ANOVA).

In the animal study, the drug was well tolerated during the experiments without evident lethal toxicity or animal body weight loss during treatment, in accordance with reported data (14). Figure 8 shows the results (means±SD) of the weight of the tumors measured for each experimental group at 1, 5, 8, 12, 15, 20, 23, and 29 days of observation.

In the absence of any treatment, the tumor showed a clear exponential growth (Figure 8A); the treatment of the animals with the vehicle, used to dissolve the drug, did not modify the tumor growth. However, the presence of RU486 significantly delayed the growth of the tumor. Inferential statistical analysis of the results indicated that a statistical reduction of the tumor volume was already present at days 8, 12, 15, and 20 of treatment and still evident at day 23, after the end of the treatment. No statistical difference (P=0.06) was observed at day 29, even if a decrease of 40% of treated tumor volume vs control vehicle was detected.

On histological examination, the control tumor (Figure 8B) showed the features of early-differentiated neuroblastoma, composed by typical neuroblasts and neuropils; several hypochromic necrotic regions were present in RU486-treated tumors (Figure 8C).

## Discussion

In the present study, the effect of the glucocorticoid/progesterone antagonist mifepristone (RU486) on SK-N-SH neuroblastoma cell line was investigated. The study demonstrated that RU486 may exert a dose-dependent significant inhibitory effect on the growth of human neuroblastoma SK-N-SH cells both *in vitro* and *in vivo*.

These results confirmed and widened our preliminary results showing that RU486 may affect the viability of SK-N-SH cells during a 9-day treatment (12); an effect not reversed by the simultaneous treatment of the cells with equal concentrations of progesterone or dexamethasone. In the present study, we confirmed that the effect of RU486 on proliferation of SK-N-SH was accompanied by a decrease of DNA synthesis (thymidine incorporation) excluding a cytotoxic action. The reduced DNA synthesis induced by RU486 was not reversed by progesterone or dexamethasone; rather, as observed on viability, RU486 seemed to exert an additive effect on the inhibitory action induced by progesterone (12). The present results are in line with the observation that high dose progesterone decreases human neuroblastoma SK-N-AS cells viability *in vitro* and that this effect is not blocked by RU486, rather a synergic effect is reported (4).

The viability of the cells was significantly inhibited at both 6 and 9 days of exposure to RU486, starting from a 3-μM concentration. The inhibitory action of RU486 on neuroblastoma cells here reported appeared similar, or more efficient, compared to that observed in other cancer cell models (22).

Just after a 6-day treatment of SK-N-SH cells with mifepristone, we observed a change in cell morphology with a prevalence of a flat epithelial-like phenotype. SK-N-SH cells are phenotypically heterogeneous and formed by at least three distinct cell types termed N, neuroblastic in appearance, S, larger and flattened resembling epithelial or fibroblast cells, and I, morphologically intermediate (23). The observed morphological changes of the cells could suggest a selective action of RU486 on the potentially more malignant N cell type (24); however, the lack of dead cells in the *in vitro* experiment would be suggestive of a reduced proliferation rate of SK-N-SH cells, with a possible interconversion from N to the less aggressive S cell type (24,25). It is accepted that the degree of cell differentiation of the tumor may be a prognostic indicator of the disease (1), therefore further investigations will be needed to clarify this hypothesis.

RU486 was found to decrease migration and invasion of glia (U87MG), ovary (SKOV-3), breast (MDA-MB231), and prostate (LNCaP) cancer cells (26). Moreover, it was also demonstrated that treatment with RU486 induces a distinct pattern of migration in these different cell lines, as well as for nuclear location during migration and the apparent redistribution of F-actin to the nucleus (26).

The actions of RU486 here described seemed linked to the chemical structure of the compound since, in contrast to the opposite effects observed for progesterone and dexamethasone, the treatment of neuroblastoma cells with the progesterone antagonist onapristone (ZK98299) shows a similar inhibitory action.

The results here described show that RU486 led to a rapid reduction of Ras-mitogen activated protein-kinase (MAPK) activity and phosphorylation in SK-N-SH cells. MAPK pathway is activated by several growth factors (e.g., PDGF, EGF) in neuroblastoma cells (27,28), where it may modulate their resistance to cytotoxic drugs (28), and the results confirmed the effect of RU486 in the regulation of growth-activated intracellular pathways. Accordingly, in other neuroblastoma cells (SH-SY-5Y), it has been reported that RU486 may antagonize the MAPK activation induced by dexamethasone/desipramine treatment (29).

Many of these observations seem to suggest an action of RU486 that is independent of its interaction with the classical progesterone/glucocorticoid receptor in SK-N-SH cells, an observation already reported in other models (1316,). For instance, RU486 may induce apoptosis in LNCaP prostate cancer line with a mechanism not antagonized by progesterone or hydrocortisone; conversely, an additive effect of the combination of RU486 with the two steroids on the increase in DNA fragmentation and Bcl2 downregulation was observed (13).

It could be hypothesized that mifepristone may exert non-genomic effects. According to this hypothesis, in our previous study we showed a unique inhibitory effect of RU486 on the binding of opiates to mu brain opioid receptors (30) due to a decreased receptor affinity, possibly linked to their uncoupling with G proteins; this evidence is suggestive of the interaction of this synthetic steroid on the transduction of intracellular signals that might be involved in controlling cell growth.

Although the molecular mechanism of RU486 on cancer cells remains to be clarified, it has been reported that RU486 may inhibit the growth of different cancer cell lines with a cytostatic effect at lower concentrations (5-10 μM) and a toxic effect at higher doses (20-40 μM) (13,22). A cytostatic effect of RU486 was also previously described in T-47D human breast cancer cells where antiprogestin reduced the proportion of cells in the S phase of the cell cycle by blocking the transition to the G1 phase (31). Actually, the data here reported seem to support possible cytostatic rather than cytotoxic effects. Moreover, it is noteworthy that the most effective concentration of RU486 that inhibits the growth of SK-N-SH cells *in vitro* (10 μM) corresponds to the range of steroid serum concentrations found in women taking the drug as abortive therapy (32), and therefore clinically applicable.

A series of studies have shown an inhibitory effect of RU486 on the growth of tumor cell xenografts in nude mice, such as meningioma (14,33), glioma (9), prostate cancer (13), ovarian cancer metastasis (26), and melanoma (8,11). In this study, we also evaluated the effect of RU486 against SK-N-SH cells xenografted *in vivo*. RU486 was administered for four weeks at a dose of 15 mg·kg^-1^·day^-1^ (corresponding to 0.3-0.5 mg/mouse), in line with previously reported doses ranging from 2.5 to 50 mg/kg (8,11,22). According to the low toxicity profile shown by RU486 as an anti-progesterone agent, in the present study, RU486 was well tolerated without evident toxicity.

A significant difference ranging from 50 to 75% of growth inhibition was observed between the RU486-treated and control mice during drug exposure and a residual 40% of inhibition was still found 9 days after the suspension of the treatment. Of interest, also in *in vivo* experiments, RU486 showed to be active at the clinically relevant human equivalent dose of 1.2 mg/kg (34).

The antiproliferative effect of RU486 in normal or neoplastic cells appears to be complex; its action was found to be dependent on the interaction with progesterone receptor in cultured human meningioma cells (35) or with glucocorticoid receptor, in prostate cancer cells (13). On the other hand, it is now common opinion that many effects of RU486, including the growth inhibition of several cancer cells, are not dependent upon expression of classical nuclear steroid receptors (16,30) or the antagonism to progesterone effects (31,36).

With regard to the effect on tumor growth, while the steroid antagonist treatment caused a significant retardation of tumor progression, a complete inhibition or prevention of tumor growth did not occur. However, RU486 could possibly act as regulator of tumor metastatic diffusion, since an inhibitory effect of the RU486 *in vivo* on cancer metastasis has been described (26). Moreover, RU486 may be used as an adjuvant or a chemo-sensitizing agent in association with more conventional anticancer drugs, as suggested by a number of reports. Actually, a quite successful association of RU486 with temozolomide, vemufarenib, docetaxel, and enzatimide has been reported (9-11).

In conclusion, in the present study, we demonstrated that RU486 may inhibit the growth of the human neuroblastoma SK-N-SH cells *in vitro* and *in vivo* by a possible cytostatic effect. This action seems to be independent of the interaction with progesterone or glucocorticoid receptors and provided some new insight on a possible new pharmacological adjuvant for the therapeutic management of an often incurable tumor.
